# Mangiferin promotes macrophage cholesterol efflux and protects against atherosclerosis by augmenting the expression of ABCA1 and ABCG1

**DOI:** 10.18632/aging.102498

**Published:** 2019-12-02

**Authors:** Kun Ren, Heng Li, Hui-Fang Zhou, Yin Liang, Min Tong, Lu Chen, Xi-Long Zheng, Guo-Jun Zhao

**Affiliations:** 1 The Sixth Affiliated Hospital of Guangzhou Medical University, Qingyuan City People’s Hospital, Qingyuan, Guangdong, China; 2 Department of Pathophysiology, School of Basic Medical Sciences, Anhui Medical University, Hefei, Anhui, China; 3 Institute of Cardiovascular Disease, Key Lab for Arteriosclerology of Hunan Province, University of South China, Hengyang, Hunan, China; 4 Department of Biochemistry and Molecular Biology, The Libin Cardiovascular Institute of Alberta, The University of Calgary, Health Sciences Center, Calgary, AB, Canada; 5 Key Laboratory of Molecular Targets and Clinical Pharmacology, School of Pharmaceutical Sciences, Guangzhou Medical University, Guangzhou, Guangdong, China; 6 Department of Histology and Embryology, Guilin Medical University, Guilin, Guangxi, China

**Keywords:** mangiferin, ABCA1/G1, LXRα, PPARγ, cholesterol efflux

## Abstract

Mangiferin has been identified as a potent cardioprotective factor that enhances high-density lipoprotein cholesterol levels in plasma. The aim of this study was to investigate the impact of mangiferin on macrophage cholesterol efflux and the development of atherosclerosis. The results showed that mangiferin injection significantly decreased atherosclerotic plaque size, and reduced plasma levels of low-density lipoprotein cholesterol, triglyceride, and total cholesterol in apoE knockout mice, whereas reverse cholesterol transport efficiency and high-density lipoprotein cholesterol levels were enhanced. *In vitro* study showed that mangiferin prevented lipid accumulation and promoted [^3^H]-cholesterol efflux from acetylated LDL-loaded RAW264.7 macrophages with an increase in the expression of ATP binding cassette A1/G1 (ABCA1/G1), liver X receptor-α (LXRα) and peroxisome proliferator-activated receptor-γ (PPARγ). Moreover, transfection of PPARγ siRNA or LXRα siRNA markedly abolished the positive effects of mangiferin on ABCA1/G1 expression and cholesterol efflux. The opposite effects were observed after treatment with PPARγ agonist rosiglitazone or LXRα agonist T0901317. In conclusion, mangiferin may attenuate atherogenesis by promoting cholesterol efflux from macrophages via the PPARγ-LXRα-ABCA1/G1 pathway.

## INTRODUCTION

Cardiovascular disease (CVD) is one of the most common causes of morbidity and death worldwide [1]. Atherosclerosis (AS), the underlying pathophysiological basis of CVD, is classed as a disease of aging and is characterized by the deposition of foam cells within the arterial wall [2]. High-density lipoprotein (HDL) possesses a variety of antiatherogenic activities, including reverse cholesterol transport (RCT), anti-inflammation, antioxidation and antithrombosis [3]. RCT, the predominant athero-protective strategy associated with HDL, is a process by which superfluous peripheral cholesterol is conveyed back to the liver for secretion into bile and feces [4]. The initial and key step of RCT is ATP-binding cassette transporter A1/G1 (ABCA1/G1)-mediated cholesterol efflux from nonhepatic peripheral tissues (e.g. macrophages and vascular smooth muscle cells) to extracellular lipid acceptors, resulting in HDL formation [5]. Tangier disease is characterized by the absence of HDL cholesterol (HDL-C) from plasma and increased susceptibility to CVD [6]. ABCA1 gene mutation and impaired removal of cellular cholesterol to apoA-1 are responsible for Tangier disease [7]. In addition, suppression of ABCG1 expression by antisense oligonucleotides can decrease phospholipid and cholesterol efflux from lipid-laden macrophages to HDL [8]. Charvet et al. [9] reported that ABCA1^-/-^/ABCG1^-/-^ mice displayed reduced cholesterol efflux from peritoneal macrophages, massive foam cell infiltration in the heart, larger proximal aortic root lesion areas and typical atherosclerotic plaques with fibrous caps compared to those of a control group. Thus, enhancement of ABCA1 and ABCG1 expression can greatly alleviate macrophage lipid deposition and atherogenesis [10].

Macrophage ABCA1/G1 expression and cholesterol efflux are modulated by a large network of factors and signaling pathways, the principle of which is the peroxisome proliferator-activated receptor-γ (PPARγ)/ liver X receptor-α (LXRα) pathway. In primary human monocyte-derived macrophages, treatment with PPARγ activators significantly enhanced the expression of ABCA1 and LXRα. Furthermore, activation of PPARγ notably amplified cholesterol efflux from THP-1-derived foam cells to apoA-1. However, pretreatment with ABCA1 inhibitor abolished PPARγ-induced cholesterol efflux, indicating that functional ABCA1 expression is necessary for PPARγ-induced cholesterol efflux from macrophages [11]. Claudel et al. demonstrated that administration of PPARγ compound or retinoid X receptor (RXR)/LXR ligands to apoE^-/-^ mice significantly reduced atherosclerotic lesion areas, the mechanisms of which involve stimulation of ABCA1-mediated cholesterol efflux [12]. Additionally, evidence has shown that LXRα can also upregulate ABCG1 expression in macrophages [13]. Therefore, activation of the macrophage PPARγ-LXRα-ABCA1/G1 pathway may be a promising therapeutic strategy against exacerbation of atheroma lesions.

Mangiferin, a xanthonoid from *Salacia oblonga*, is beneficial for the maintenance of glucolipid metabolism homeostasis. Muruganandan et al. [14] determined that chronic intraperitoneal administration of mangiferin in diabetic mice resulted in a drastic reduction in plasma glucose, triglyceride (TG), total cholesterol (TC) and low-density lipoprotein cholesterol (LDL-C) levels and an elevation in HDL-C concentration, indicating its powerful antihyperlipidemic and antiatherogenic activities. Similarly, another study by Na et al. [15] showed that in overweight patients with hyperlipidemia, participants receiving mangiferin exhibited reduced serum TG and free fatty acid (FFA) levels and insulin resistance index, while plasma HDL-C levels and lipoprotein lipase (LPL) activity were increased. Importantly, mangiferin inhibits hypercholesterolemia and inflammation through PPARγ activation. Treatment of diabetic mice with mangiferin dramatically increased serum HDL-C levels and decreased glucose, TG, TC, very low-density lipoprotein cholesterol (VLDL-C), and LDL-C levels via dual activation of PPARγ/glucose transporter type 4 (GLUT4) signaling pathways [16]. In high-fat diet (HFD)-fed obese rats, injection of mango leaf tea, the main component of which is mangiferin, markedly downregulated serum TG and TC levels, with obvious amplification of PPARγ expression in adipose tissue [17]. In addition, Qu et al. [18] identified that mangiferin diminished interleukin-1β (IL-1β)-induced NF-κB activation and production of matrix metalloproteinase-1 (MMP-1) and MMP-3 in human osteoarthritis chondrocytes by activating PPARγ. In this paper, we designed experiments to elucidate whether mangiferin exerts hypolipidemic effects via stimulation of the PPARγ-LXRα-ABCA1/G1 pathway, which likely mitigates atheromatous plaque formation in apoE^-/-^ mice.

## RESULTS

### Mangiferin treatment attenuates atheromatous plaque formation and improves serum lipid profiles in apoE^-/-^ mice

**Figure 1:**
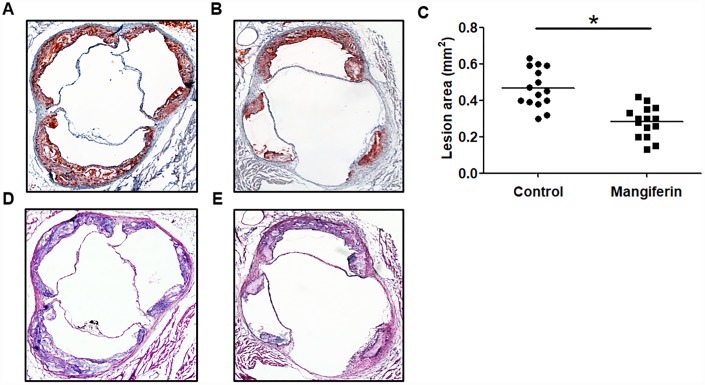
**Mangiferin reduces atherosclerotic lesion formation in apoE^-/-^ mice.** Eight-week-old male apoE^-/-^ mice were intraperitoneally injected with PBS or mangiferin (15 mg/kg) every day for 12 weeks. (**A**–**B**) Representative image of Oil Red O staining of an aortic lesion. Original magnification: 40×. (**C**) Quantification of the lesion areas of mice (n=15/group). *P < 0.05 *vs.* control group. Values are expressed as the mean ± SEM (n =15/group). (**D**–**E**) Representative HE staining of an aortic lesion in apoE^-/-^ mice. Original magnification: 40×.

To explore the effects of mangiferin on AS development *in* *vivo*, apoE^-/-^ mice fed an HFD were intraperitoneally injected with mangiferin every day for 12 weeks. The results showed that mangiferin injection dramatically attenuated atherosclerotic lipid accumulation (Figure 1A–1C) and lesion size (Figure 1D and 1E) and increased collagen content (Supplementary Figure 1) in aortic roots compared with those of control mice. Moreover, as shown in Table 1, TG, TC and LDL-C serum levels were greatly reduced, while plasma HDL levels were elevated after treatment with mangiferin. These observations suggest that mangiferin improves serum lipid profiles and inhibits AS progression *in vivo*.

**Table 1:** Bodyweight and plasma lipid profile in apoE^-/-^ mice.

	**Control (n=15)**	**Mangiferin (n=15)**
Body weight (g)	28.42 ± 2.37	29.36 ± 3.24
TG (mmol/L)	1.78 ± 0.34	1.17 ± 0.29*
TC (mmol/L)	18.52 ± 2.23	14.73 ± 1.36*
HDL-C (mmol/L)	1.39 ± 0.17	2.54 ± 0.31*
LDL-C (mmol/L)	14.57 ± 1.95	10.05 ± 1.84*

TC, total cholesterol; TG, triglyceride; HDL-C, high-density lipoprotein cholesterol; LDL-C, low-density lipoprotein cholesterol. *P < 0.05 *vs*. control group.

### Mangiferin increases RCT *in vivo* and accelerates cholesterol efflux from RAW264.7 macrophages

**Figure 2:**
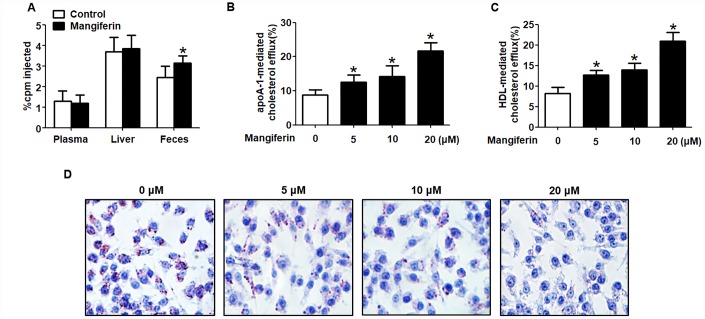
**Mangiferin promotes macrophage cholesterol efflux and enhances RCT in apoE^-/-^ mice.** (**A**) [^3^H]-cholesterol-labeled RAW264.7 cells were intraperitoneally injected into apoE^-/-^ mice. The amounts of [^3^H]-tracer in the liver, feces and plasma were assessed by LSC. *P < 0.05 *vs.* control group. (**B**–**D**) RAW264.7 macrophage-derived foam cells were treated with mangiferin at different concentrations (0, 5, 10, and 20 μM) for 24 h. Then, the percent cholesterol efflux to apoA-1 (**B**) or HDL (**C**) was analyzed by LSC. Lipid droplet content was assessed using Oil Red O staining (**D**). All results are presented as the mean ± SEM from three independent experiments, each performed in triplicate. *P < 0.05 *vs.* 0 μM group.

Given that the progression of AS is closely related to an impaired RCT rate [19], we further determined whether mangiferin-induced athero-protection is attributed to stimulation of RCT. ApoE^-/-^ mice were intraperitoneally injected with [^3^H]-cholesterol- labeled RAW264.7 macrophages. Then, [^3^H]-labelled cholesterol levels in plasma, liver and feces were measured to assess cholesterol distribution along the RCT pathway by liquid scintillation counting (LSC). The results showed that [^3^H]-cholesterol counts in plasma and liver did not differ markedly, while [^3^H]-cholesterol tracer amounts in feces were markedly amplified in mangiferin-treated mice compared with those of the control group (Figure 2A). These results are consistent with the cholesterol mass in plasma lipoprotein distribution, namely, increased HDL levels and decreased LDL in mangiferin-treated mice, demonstrating that mangiferin promotes macrophage-to-feces RCT *in vivo*.

Since cholesterol efflux from macrophage foam cells is regarded as the first and critical step of RCT [20, 21], we next explored the effects of mangiferin on macrophage cholesterol efflux *in vitro*. RAW264.7 cells were fully differentiated and then exposed to different concentrations of mangiferin (0, 5, 10, and 20 μM) for 24 h. Percent cholesterol efflux, lipid droplet accumulation and intracellular cholesterol content were assessed by LSC, Oil Red O staining and high-performance liquid chromatography (HPLC), respectively. The MTT assay showed that mangiferin treatment did not exert cytotoxic effects on RAW264.7 macrophage-derived foam cells (Supplementary Figure 2). Moreover, mangiferin potently magnified apoA-1- or HDL-mediated [^3^H]-cholesterol efflux (Figure 2B, 2C), reduced cellular lipid droplet accumulation (Figure 2D), and decreased TC, free cholesterol (FC), and cholesteryl ester (CE) content (Table 2) in a dose-dependent manner. Taken together, these data indicate that mangiferin enhances macrophage cholesterol efflux and increases *in vivo* RCT efficiency.

**Table 2:** Effects of different concentrations of mangiferin on cholesterol content in RAW264.7 macrophage-derived foam cells.

**Mangiferin (μM)**	**0**	**5**	**10**	**20**
TC (mg/g)	491 ± 25	345 ± 16*	318 ± 21*	198 ± 18*
FC (mg/g)	192 ± 22	139 ± 18*	121 ± 13*	84 ± 15*
CE (mg/g)	299 ± 19	206 ± 14*	197 ± 17*	114 ± 11*
CE/TC (%)	60.9	59.7	61.9	57.6

TC: total cholesterol; FC: free cholesterol; CE: cholesteryl ester; * compared with control group: *P* < 0.05.

### Mangiferin induces the expression of ABCA1/G1 in RAW264.7 macrophage-derived foam cells

**Figure 3:**
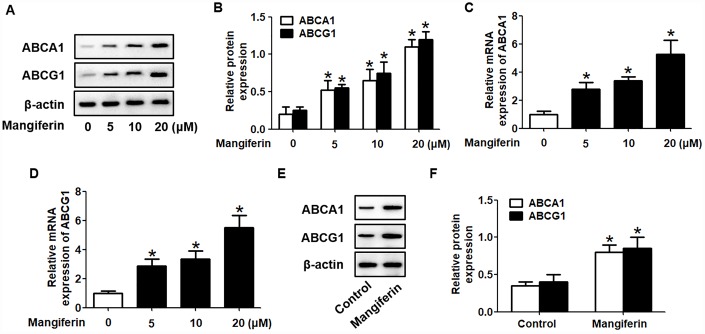
**Mangiferin promotes the expression of ABCA1 and ABCG1 in RAW264.7 macrophages and in the aortas of apoE^-/-^ mice.** (**A**–**D**) RAW264.7 macrophage-derived foam cells were exposed to different concentrations of mangiferin (0, 5, 10, and 20 μM) for 24 h. Then, the protein (**A**, **B**) and mRNA (**C**, **D**) levels of ABCA1 and ABCG1 were determined by western blot and RT-qPCR analyses, respectively. *P < 0.05 *vs.* 0 μM group. (**E**–**F**) The mice were divided and treated as described above. The protein levels of ABCA1 and ABCG1 in the homogenate of the aortic arch were detected by western blotting. *P < 0.05 *vs.* control group. Data are presented as the mean ± SEM (n =3/group).

ABCA1 and ABCG1 are two key players in cholesterol efflux from foam cells and the *in vivo* RCT pathway [22]. To determine the underlying mechanisms by which mangiferin promotes cholesterol efflux and RCT, we investigated the effect of mangiferin on the expression of ABCA1/G1. RAW264.7 macrophage-derived foam cells were treated with various concentrations of mangiferin (0, 5, 10, and 20 μM) for 24 h and then harvested for western blot and RT-qPCR analyses. The results showed that mangiferin potently enhanced the protein and mRNA levels of ABCA1/G1 in a concentration- dependent manner (Figure 3A–3D). In addition, the protein levels of ABCA1/G1 were increased in the aortic roots of mangiferin-injected mice compared with those of the control mice (Figure 3E, 3F). Moreover, mangiferin treatment did not significantly influence the degradation and phosphorylation of ABCA1/G1 protein (Supplementary Figure 3). Therefore, the stimulating effects of mangiferin on macrophage cholesterol efflux and *in vivo* RCT are likely achieved by amplification of ABCA1/G1 expression.

### Role of the PPARγ/LXRα pathway in the effects of mangiferin on ABCA1/G1 expression in and cholesterol efflux from RAW264.7 macrophage-derived foam cells

**Figure 4:**
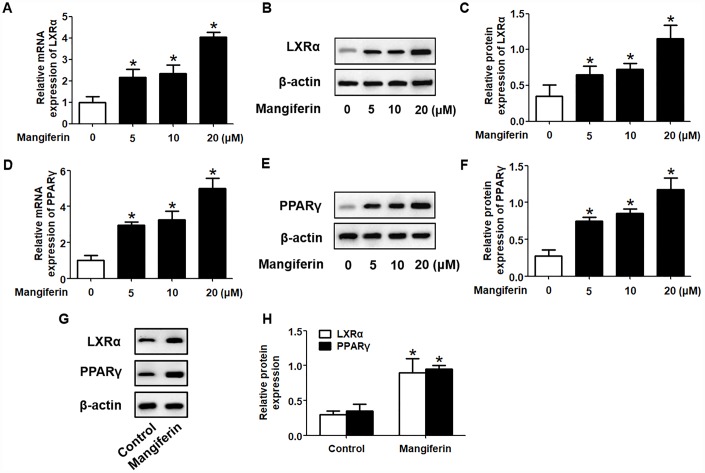
**Mangiferin enhances the expression of PPARγ and LXRα in RAW264.7 macrophages and in the aortas of apoE^-/-^ mice.** (**A**–**F**) After RAW264.7 cells were fully differentiated, the cells were exposed to different concentrations of mangiferin (0, 5, 10, and 20 μM) for 24 h. Then, RT-qPCR and western blot analyses were performed to detect the mRNA (**A**, **D**) and protein (**B**, **C**, **E**, **F**) levels, respectively, of PPARγ and LXRα. *P < 0.05 *vs.* 0 μM group. (**G**, **H**) Protein levels of PPARγ and LXRα in the homogenate of the aortic arch were assessed by western blotting. *P < 0.05 *vs.* control group. All results were collected from three independent experiments, each performed in triplicate. Data are presented as the mean ± SEM (n =3/group).

**Figure 5:**
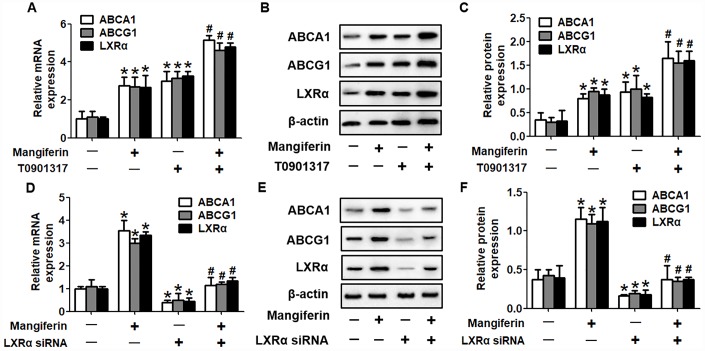
**Mangiferin promotes ABCA1 and ABCG1 expression by upregulating LXRα levels.** RAW264.7 macrophage-derived foam cells were pretreated with 2 μM T0901317 (**A**–**C**) or transfected with 20 nM LXRα siRNA (**D**–**F**) and then incubated with mangiferin (20 μM) for 24 h. RT-PCR and western blot analyses were performed to assess the mRNA and protein levels, respectively, of ABCA1/G1 and LXRα. All results were obtained from three independent experiments, each performed in triplicate. Data are expressed as the mean ± SEM (n =3/group). *P < 0.05 *vs*. control group; ^#^*P* < 0.05 *vs.* mangiferin only group.

**Figure 6:**
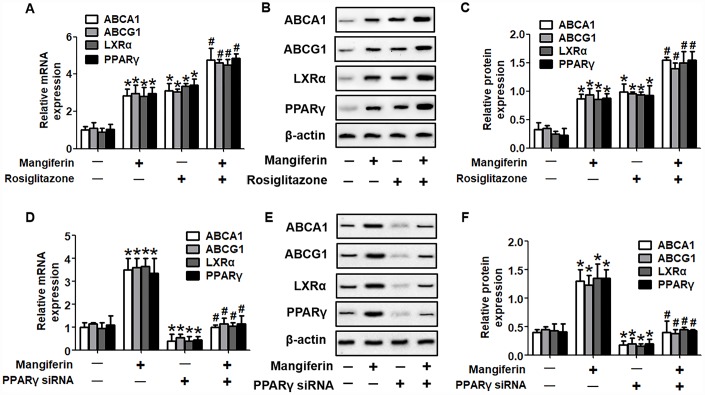
**PPARγ mediates the positive effects of mangiferin on the expression of ABCA1/G1 and LXRα.** RAW264.7 macrophage-derived foam cells were pretreated with 25 μM rosiglitazone (**A**–**C**) or transfected with 20 nM PPARγ siRNA (**D**–**F**) and then incubated with mangiferin (20 μM) for 24 h. RT-PCR and western blot analyses were performed to assess the mRNA and protein levels, respectively, of ABCA1/G1, LXRα and PPARγ. All results were obtained from three independent experiments, each performed in triplicate. Data are expressed as the mean ± SEM (n =3/group). *P < 0.05 *vs*. control group; ^#^*P* < 0.05 *vs.* mangiferin only group.

**Figure 7:**
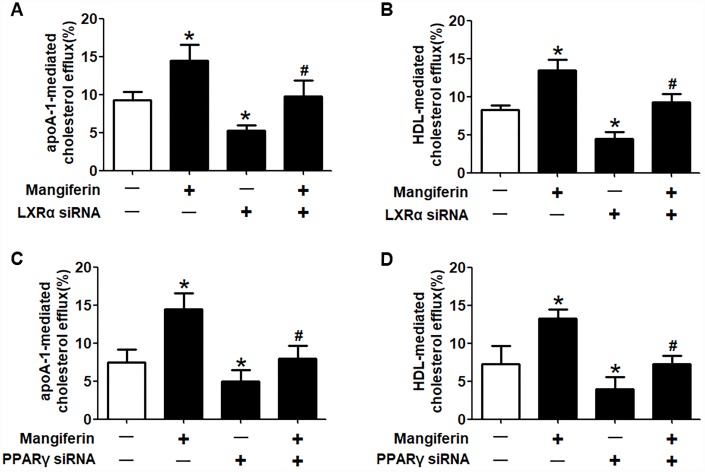
**Mangiferin promotes cellular cholesterol efflux through the upregulation of PPARγ and LXRα expression.** RAW264.7 macrophage-derived foam cells were pretreated with LXRα siRNA (**A**, **B**) or PPARγ siRNA (**C**, **D**) and subsequently treated with 20 μM mangiferin for 24 h. LSC assays were performed to detect apoA-1- or HDL-mediated [^3^H]-cholesterol efflux. All results were obtained from three independent experiments, each performed in triplicate. Data are presented as the mean ± SEM (n =3/group). *P < 0.05 *vs*. control group; ^#^*P* < 0.05 *vs.* mangiferin only group.

It is well known that the PPARγ/LXRα pathway is critical in modulating macrophage ABCA1/G1 expression and lipid homeostasis [23]. We next examined whether the PPARγ/LXRα pathway is implicated in the positive effects of mangiferin on ABCA1/G1 expression and cholesterol efflux. As shown in Figure 4A–4F, treatment of RAW264.7 macrophage-derived foam cells with mangiferin markedly elevated the mRNA and protein expression of LXRα and PPARγ in a dose-dependent manner. Similarly, in the aortic roots of mangiferin-injected mice, the protein levels of LXRα and PPARγ were also increased compared with those of the control group (Figure 4G, 4H), indicating that mangiferin elevates LXRα and PPARγ expression both *in vitro* and *in vivo*. Additionally, treatment of foam cells with the LXRα agonist T0901317 significantly magnified mangiferin-induced ABCA1/G1 mRNA and protein expression (Figure 5A–5C). In contrast, transfection of LXRα siRNA abolished the positive effects of mangiferin on the expression of ABCA1/G1 (Figure 5D–5F), demonstrating that LXRα mediates the stimulatory effect of mangiferin on ABCA1/G1 expression. Next, we further confirmed whether PPARγ is involved in the effects exerted by mangiferin. As shown in Figure 6A–6C, incubation with rosiglitazone, a selective PPARγ agonist, notably amplified the mangiferin-induced mRNA and protein levels of ABCA1/G1 and LXRα. The opposite effects were observed after transfection with PPARγ siRNA (Figure 6D–6F), indicating that mangiferin promotes ABCA1/G1 expression via activation of the PPARγ/LXRα pathway. Furthermore, transfection of LXRα siRNA or PPARγ siRNA also substantially compensated for mangiferin-induced cellular cholesterol efflux from foam cells to apoA-1 or HDL (Figure 7). Taken together, these results suggest that mangiferin exerts its positive effects on ABCA1/G1 expression and cholesterol efflux, at least in part, via activation of the PPARγ/LXRα pathway in RAW264.7 macrophage-derived foam cells.

## DISCUSSION

Atherosclerotic plaque initiation and progression are characterized by the massive deposition and accumulation of lipid-loaded macrophages within arterial walls [24]. Disturbed cholesterol-handling machinery in macrophages, especially impaired cholesterol efflux capacity, correlates closely with foam cell formation, aberrant serum lipid profile and reduced RCT efficiency, contributing to atherogenesis [25]. Recently, many food and herbal extracts have been identified as beneficial factors in preventing AS [26, 27]. In the present study, we investigated the effects of mangiferin on atheromatous plaque formation in apoE^-/-^ mice and ABCA1/G1-mediated cholesterol efflux from macrophage foam cells and the underlying mechanisms. The results showed that mangiferin administration dramatically reduced intraplaque lipid accumulation, decreased atheromatous lesion areas and increased collagen content in aortic roots of apoE^-/-^ mice. In addition, mangiferin treatment also enhanced RCT efficiency and improved plasma lipid profiles *in vivo*. Furthermore, incubation of macrophages with mangiferin significantly amplified ABCA1/G1 expression and ABCA1/G1-mediated cholesterol efflux and reduced intracellular cholesterol content in macrophage foam cells. Finally, we demonstrated that the PPARγ/LXRα signaling pathway is involved in mangiferin-induced ABCA1/G1 expression and athero-protective effects.

Mangiferin is known as a natural polyphenol isolated from mango fruit and its byproducts (i.e., peel and kernel), and it possesses potent antiapoptotic, anti-inflammatory and antioxidant properties [28–30]. In recent years, evidence has indicated an inverse relationship between mangiferin and AS progression [14, 31]. While mangiferin can exert strong antihyperlipidemic effects by increasing HDL-C levels and decreasing TG, TC, VLDL-C and LDL-C levels in the plasma, the potential molecular mechanisms have not been investigated. Given that ABCA1/G1-mediated cholesterol efflux from peripheral cells plays a critical role in HDL formation and the RCT pathway, we inferred that the hypolipidemic activities of mangiferin may be mediated by ABCA1/G1. After treatment of RAW264.7 macrophage-derived foam cells with mangiferin, ABCA1/G1 expression and percent cholesterol efflux to apoA-1 or HDL were markedly enhanced. In atherosclerotic mice, mangiferin injection likewise promoted RCT efficiency and improved serum cholesterol profiles, with amplification of aortic ABCA1/G1 expression. These findings confirm our hypothesis and are consistent with previous observations.

LXRα, identified as a transcriptional activator, plays a key role in modulating glycolipid metabolism and maintaining cholesterol homeostasis [32]. Research has shown that ABCA1 and ABCG1 are the target genes of LXRα and that LXRα activation directly enhances ABCA1/G1 expression [33, 34]. Zeng et al. [35] observed that dihydromyricetin (DMY), a bioactive flavonoid component abundant in the leaves of *Ampelopsis grossedentata*, upregulates NBD-cholesterol efflux to apoA-1 or HDL and attenuates oxidized (ox)-LDL-induced lipid deposition in human THP-1-derived macrophages via activation of the LXRα-ABCA1/ABCG1 pathway, which further increased serum HDL-C levels and inhibited atherosclerotic plaque formation. Similarly, Jin et al. [36] reported that homocysteine (Hcy), a nonessential amino acid, exacerbated aortic lesion development and intraplaque lipid accumulation in apoE^-/-^ mice by decreasing cholesterol efflux from THP-1 macrophage-derived foam cells through inhibition of the LXRα-ABCA1/ABCG1 signaling pathway. Furthermore, treatment of mice with LXRα agonist significantly compensated for Hcy-induced lesion area and lipid accumulation in aortic plaques of apoE^-/-^ mice, thus weakening the atherogenic effects exerted by Hcy. 9-cis-retinoic acid (9-cis-RA) can increase serum HDL-C concentrations and reduce atherosclerotic lesion areas *in vivo*. Treatment of J774A.1 macrophages with 9-cis-RA significantly enhanced the expression of ABCA1/G1 and LXRα, cholesterol efflux, and alleviated lipid accumulation, effects that were suppressed by LXRα knockdown [37]. In the current study, we observed that macrophage LXRα expression levels were dramatically increased by mangiferin treatment in a concentration-dependent manner and that incubation with LXRα siRNA abrogated mangiferin-stimulated ABCA1/G1 expression, as well as intracellular cholesterol efflux. Furthermore, LXRα protein levels were also increased in aortic roots of mangiferin-injected apoE^-/-^ mice compared to those of the control group. These outcomes indicated that LXRα is implicated in mangiferin-induced cholesterol unloading and athero-protection.

PPARγ belongs to the nuclear receptor superfamily, members of which are ligand-inducible transcription factors that modulate various pathways involved in the development of diabetes, obesity and AS [38]. PPARγ is highly expressed in macrophages and acts as a central switch that controls macrophage inflammation, polarization and lipid metabolism in atherosclerotic plaques [39–41]. Chawla and colleagues [42] showed that PPARγ and LXRα worked together to promote ABCA1/G1 expression and cholesterol efflux from lipid-laden macrophages. Moreover, they demonstrated that the positive effects of PPARγ ligands (rosiglitazone and GW7845) on ABCA1/G1-mediated lipid efflux were secondary to the induction of LXRα expression, which was completely inhibited in PPARγ^-/-^ macrophages. The group further verified that only PPARγ/RXRα but not PPARα or PPARβ specifically bound to the DR-1 PPAR response element (PPRE) on the LXRα promoter, thus directly regulating LXRα expression. Additionally, atherosclerotic mice transplanted with PPARγ^-/-^ bone marrow (PPARγ^-/-^ BMT) displayed more severe lipid accumulation and larger lesion areas in aortic valves compared to those of the PPARγ^+/+^ BMT recipients. Another study showed that pioglitazone, a PPARγ agonist, transcriptionally stimulated ABCA1/G1 expression and enhanced apoA-1- or HDL-mediated cholesterol efflux from human THP-1 cells and mouse RAW264.7 macrophages in an LXRα-dependent manner [43]. Gu et al. [44] showed that in atherosclerotic mice induced by chronic unpredictable mild stress (CUMS), the protein levels of PPARγ, LXRα, and ABCA1 in aorta were significantly decreased. Moreover, RAW264.7 macrophages under CUMS displayed increased intracellular lipid accumulation and reduced expression of PPARγ, LXRα, and ABCA1, effects that could be abolished by treatment with PPARγ agonist. Chlorogenic acid (CGA), another kind of abundant polyphenol in daily food, can markedly diminish atherosclerotic lesion sizes in the valve areas of aortic roots, the plasma levels of TC, TG and LDL-C and serum concentrations of various proinflammatory cytokines. *In vitro* experiments showed that CGA exerts strong stimulating effects on the expression of PPARγ, LXRα, ABCA1 and ABCG1, as well as cholesterol efflux from RAW264.7 macrophages to apoA-1 or HDL [45]. These studies demonstrated the crucial role of PPARγ in inducing LXRα and ABCA1/G1 expression, preventing foam cell formation and AS progression. Intriguingly, Jiang et al. [46] reported that PPARγ ligands, especially troglitazone, markedly reduced LXRα ligand-stimulated ABCA1 expression and apoA-1-mediated cholesterol efflux from wild type and CD36^-/-^ peritoneal macrophages, while ABCG1 expression and HDL-mediated cholesterol efflux were significantly increased. The molecular mechanisms underlying these contradictory observations warrant further exploration. In the present study, we elucidated that mangiferin promoted PPARγ expression in cholesterol-loaded RAW264.7 macrophages and in aortic roots of apoE^-/-^ mice. Furthermore, transfection of foam cells with PPARγ siRNA abolished mangiferin-induced LXRα and ABCA1/G1 expression, as well as reduced the percent cholesterol efflux, indicating that the role of mangiferin in protecting against atherogenesis is mediated by activation of the PPARγ-LXRα-ABCA1/G1 signaling pathway. However, whether other pathways and/or crosstalk effects are involved in the antiatherogenic effects of mangiferin remain to be solved.

Taken together, for the first time, our study provides new insights for mangiferin-mediated athero-protection by stimulating macrophage cholesterol efflux and alleviating lipid deposition via induction of the PPARγ-LXRα-ABCA1/G1 pathway. These findings may provide a novel angle from which to examine the therapeutic effects of mangiferin in preventing AS.

## MATERIALS AND METHODS

### Mice and treatments

Eight-week-old male apoE^-/-^ mice were purchased from Nanjing CAVENS Biological Technology Co., Ltd. and housed in an environmentally controlled room (22–26°C, 50% humidity) under 12-h light/dark cycles with free access to drinking water and food. After the animals were fed a chow diet for 2 weeks, they were randomly separated into a mangiferin group and a control group (n=15/group), and both groups were fed an HFD (containing 10% fat oil, 2% cholesterol, 4% whole milk powder and 0.5% sodium cholate) for 12 weeks. When the HFD started, mice in the mangiferin group were intraperitoneally injected with 15 mg/kg of mangiferin every day. The control group was administered an equivalent volume of phosphate-buffered saline (PBS). At week 14, the mice were sacrificed, and blood and tissue samples were taken for further assessment.

The animal experiments strictly adhered to the Guide for the Care and Use of Laboratory Animals released by the US National Institutes of Health (NIH publication no. 85–23, revised in 1996), as well as care guidelines for the use of experimental animals from Anhui Medical University. The investigation procedure was approved by the Animal Ethics Committee of Anhui Medical University. Sodium pentobarbital anesthesia was performed throughout all surgeries to minimize suffering.

### Aortic lesion assessment

The hearts and proximal aortas were dissected, perfused with PBS and fixed in 10 ml 4% buffered paraformaldehyde for 4 h. Then, specimens were soaked in PBS for 1 h and placed in 30% sucrose overnight. The following day, the hearts were embedded in optimal cutting temperature (O.C.T.) medium and stored at −20°C. Serial 8-μm sections were cut from the aortic sinus using a cryostat microtome and placed on glass slides. At least ten sections of the aortic root per mouse were analyzed. Each consecutive slide was stained with Oil Red O for assessment of lipid deposition. Every third slide from the serial sections was stained with HE and Masson’s trichrome for detection of lesion size and collagen content, respectively. Aortic plaque areas in apoE^-/-^ mice were quantified using Image Pro Plus software (Media Cybnetics, Silver Spring, MD). Data are expressed as lesion size ± SEM [47].

### Cell culture and foam cell formation evaluation by Oil Red O staining

Mouse RAW264.7 macrophages were obtained from the Type Culture Collection of the Chinese Academy of Sciences (Shanghai, China) and cultured in RPMI-1640 medium supplemented with 10% fetal bovine serum (FBS) and 2% penicillin/streptomycin in 6-well plates (5% CO_2_, 37°C). To induce foam cell formation, the cells were incubated with acetylated-low density lipoprotein (ac-LDL, 100 μg/ml) in serum-free RPMI-1640 medium containing 0.2% bovine serum albumin (BSA) for 48 h. Oil Red O staining for estimation of foam cell formation was conducted as described previously [48]. Briefly, the cells were fixed in 4% paraformaldehyde solution for 10 min and washed in 60% isopropanol for 15 s. Next, the cells were stained with filtered Oil Red O working solution at 37°C for 5 min in the dark and then destained with 60% isopropanol for 15 s. After rinsing with PBS, the cells were counterstained with HE for 5 min. A light microscope (Olympus) was used to observe the positively stained cells (red) and acquire images.

### MTT assay

To test the cell viability, a 3-(4, 5-dimethylthiazol-2-yl)-2, 5-diphenyltetrazolium bromide (MTT) assay was used. RAW264.7 macrophage-derived foam cells were seeded into a 96-well culture plate. Then, the cells were treated with mangiferin (0, 5, 10, and 20 μM) for 24 h, followed by incubation with 0.5 mg/mL of MTT at 37 °C for 4 h. A 96-well microplate autoreader (Bio-Tek Instruments Inc., Winooski, VT, USA) was used to measure the absorbance at a wavelength of 490 nm. Independent experiments were performed in triplicate.

### *In vivo* RCT assay

Macrophage-to-feces RCT was performed as described previously [49]. RAW264.7 macrophages were radiolabeled with 5 μCi/ml [^3^H]-cholesterol (PerkinElmer, Waltham, MA) and loaded with 100 μg/ml ac-LDL for 48 h. The cells were washed, equilibrated and resuspended in warm PBS to 8–12×10^6^ cells/ml. Then, a small aliquot (~50 μl) of cells was removed to measure specific [^3^H]-cholesterol activity by LSC. Subsequently, cells were drawn into individual 1 ml syringes (500 μl cells/syringe) using 25 Ga needles within 30 min after collection. Finally, 6×10^6^ labeled cells containing 8×10^5^ counts per minute (CPM) in 0.5 ml PBS were intraperitoneally injected into each apoE^-/-^ mouse.

Blood samples were taken at 24 and 48 h after injection using tubes containing EDTA via the facial vein and then centrifuged at 1500 rpm for 30 min at 4°C. Radioactive counts in plasma (~50–100 μl/mouse) were measured by LSC. Feces were continuously collected from cages until the end of the study and dissolved in an equal volume of ethanol. Triplicate aliquots of 200 μl of feces homogenate were used to measure [^3^H]-cholesterol radioactivity by LSC. At 48 h after injection, the mice were exsanguinated. Livers were collected and stored at −80°C. Frozen liver specimens were ground to a powder and subsequently homogenized in distilled water (1 ml), 500 μl aliquots of which were used for radioactivity assessment.

### Cellular cholesterol efflux assays

RAW264.7 macrophages were seeded into 6-well plates (1×10^6^ cells/well), radiolabeled with 5 μCi/ml [^3^H]-cholesterol and loaded with 100 μg/ml ac-LDL for 48 h in media containing 0.2% BSA. Afterward, equilibrated [^3^H]-cholesterol-labeled cells were washed with fresh media and subsequently treated as indicated in the figures. Then, the cells were rinsed again with PBS and incubated in the presence of apoA-1 (10 μg/ml) or HDL (50 μg/ml) for 24 h. Medium and cell-associated [^3^H]-cholesterol were then determined by LSC. Finally, the percent efflux was calculated using the following equation: [total media counts / (total cellular counts + total media counts)] × 100%.

### Real-time quantitative polymerase chain reaction (RT-qPCR)

Cellular total RNA was extracted using TRIzol reagent (Invitrogen) in accordance with the manufacturer’s protocol. A Nanodrop 3000 (Thermo Fisher) was used to assess the purity and concentration of the extracted RNA. Then, RNA (1 μg) was converted into cDNA by using a TaqMan™ reverse transcription reagent kit (Applied Biosystems). Quantitative PCR with SYBR™ green detection chemistry was performed on a StepOnePlus™ real-time PCR system (Applied Biosystems). The sequences of the real-time PCR primers used are as follows: ABCA1 sense, 5′-CGTTTCCGGGAAGTGTCCTA-3′ and antisense, 5′-GCTAGAGATGACAAGGAGGATGGA-3′; ABCG1 forward, 5′-AGGTCTCAGCCTTCTAAAGTTCCTC-3′ and reverse, 5′-TCTCTCGAAGTGAATGAAATTTAT CG-3′; PPARγ sense, 5′-CACAATGCCATCAGGTTT GG-3′ and antisense, 5′-GCTGGTCGATATCACTGGA GATC-3′; LXRα forward, 5′-GCCGAGTTTGCCTTG CTCA-3′ and reverse 5′-TCCGGAGGCTCAACCAGT TTC-3′; and β-actin sense, 5′-TGGCACCCAGCACA ATGAA-3′ and antisense, 5′-CTAAGTCATAGTCCG CCTAGAAGCA-3′. The specificity of all PCR products was assessed by melting curve analysis. Relative gene expression was analyzed using the 2^-ΔΔCt^ method and normalized against β-actin as the internal control.

### Western blot analysis

RAW264.7 macrophages and murine tissues were lysed for protein extraction using radioimmunoprecipitation assay (RIPA) buffer and phenylmethylsulfonyl fluoride (PMSF; Solarbio Life Sciences, Beijing, China) (94:6). A BCA assay kit (CWBIO, Peking, China) was used to determine the protein concentration. Proteins (20 μg per lane) were then separated with 8% gels using sodium dodecyl sulfate-polyacrylamide gel electrophoresis (SDS-PAGE, Solarbio Co., Peking, China) (120 V, 90 min). Subsequently, related proteins were transferred to 0.45 μm polyvinylidene fluoride membranes (PVDF, Merck Millipore, Darmstadt, Germany). Protein transfer efficiency was tested by Li Chunhong S staining (CWBIO, Peking, China). Thereafter, the membranes were blocked using 5% fat-free dry milk dissolved in Tris-buffered saline with Tween-20 (TBS-T) at 4°C for 4 h and then immunoblotted with primary antibodies (diluted 1:1000) against ABCA1, ABCG1, LXRα, PPARγ and β-actin (Abcam, Cambridge, UK) overnight at 4°C with gentle shaking. The next day, the membranes were rinsed three times with TBS-T (10 min each) and further incubated with horseradish peroxidase-conjugated secondary antibody (diluted 1:5000, CWBIO, Peking, China) for 2 h at room temperature. Finally, the protein bands were visualized by enhanced chemiluminescence (ECL; Merck Millipore, Darmstadt, Germany), and Quantity One software was used to quantify the relative protein levels.

### Small interfering RNA transfection

Specific small interfering RNAs (siRNAs) against PPARγ (sense, 5′-GGAUGCAAGGGUUUCUUCCTT-3′; antisense, 5′-GGAAGAAACCCUUGCAUCCTT-3′) and LXRα (sense, 5′-GGCUGCAAGUGGAAUUCAU TT-3′ and antisense, 5′-AUGAAUUCCACUUGCAGC CTT-3′) were synthesized by the GenePharma Company (Shanghai, China). Macrophages (60%-80% confluent monolayer) were seeded in 12-well plates with 1 ml of standard medium. The following day, the cells were transfected with siRNA duplexes (20 nM final concentration) using Lipofectamine™ RNAiMAX reagent (Invitrogen) according to the manufacturer’s instructions. After 72 h, RT-qPCR and western blot analyses were performed to determine transfection efficiency.

### Serum lipid analyses

ApoE^-/-^ mice were fasted overnight and euthanized. Blood samples were collected from the retro-orbital venous plexus. Plasma LDL-C, HDL-C, TG and TC levels were detected by enzymatic methods using a commercial assay kit (Nanjing Jiancheng Bioengineering Institute, China).

### Cholesterol content assay by HPLC

Following a series of rinses with PBS, 1 ml of 0.5% NaCl to 100–200 μg cellular proteins (per ml) were added to the cells. The cells were sonicated using an ultrasonic processor for 3 min, and a BCA kit was used to measure protein concentration in cell lysates. An equal amount of fresh, cold KOH (diluted with 150 g/L ethanol) was added. After supplementation with an equivalent amount of isopropanol: hexane (2:3 v/v), the mixture was vortexed and centrifuged. A 0.1 ml aliquot of cell solution (containing approximately 5–20 μg protein) was used to measure TC, and another aliquot was used for the detection of FC, which was then dissolved in isopropanol (1 mg cholesterol/ml) and stored at −20°C as a stock solution.

Ten microliters of reaction mixture (containing 5% NaCl, 500 mM Tris-HCl (pH 7.4), 500 mM MgCl_2_ and 10 mM dithiothreitol) was added to 0.1 ml of each sample. Then, each tube was supplemented with 0.4 U cholesterol oxidase in 10 μl 0.5% NaCl for FC detection, or 0.4 U cholesterol oxidase plus 0.4 U of cholesterol esterase for TC measurement. The reaction in each tube was performed at 37°C for 30 min and stopped by adding 100 μl of ethanol: methanol (1:1 v/v). Proteins were precipitated under ice-cold conditions for 20 min and then centrifuged (1500 rpm, 15°C, 10 min). Ten microliters of the supernatant were collected and analyzed with a chromatography system (PerkinElmer Inc.), including a PerkinElmer Series 600 LINK, a PerkinElmer Series 200 UV/Vis detector, a PerkinElmer Series 200 vacuum degasser, a pump and a Discovery C-18 HPLC column (Supercool Inc.). Finally, column chromatography was conducted using isopropanol:n-acetonitrile: heptane (35:52:13) at a flow rate of 1 ml/min for 10 min. Data were analyzed via TotalChrom software from PerkinElmer, and absorbance at 216 nm was monitored [50].

### Statistical analysis

All data were collected from at least three independent experiments and are shown as the mean ± standard error of the mean (SEM). A comparison of mean values was conducted using one-way ANOVA followed by Student-Newman-Keuls (SNK) post hoc test via GraphPad Prism 6 software. A *P* value less than 0.05 was considered statistically significant.

## Supplementary Material

Supplementary Figures
