# Supplementary material for: Mangiferin promotes macrophage cholesterol efflux and protects against atherosclerosis by augmenting the expression of ABCA1 and ABCG1
Source: Aging (Albany NY). 2019 Dec 2;11(23):10992–1009. doi: 10.18632/aging.102498 (PMC6932905; doi:10.18632/aging.102498)
Supplement: Supplementary Figures [file aging-11-102498-s001..pdf]

SUPPLEMENTARY FIGURES

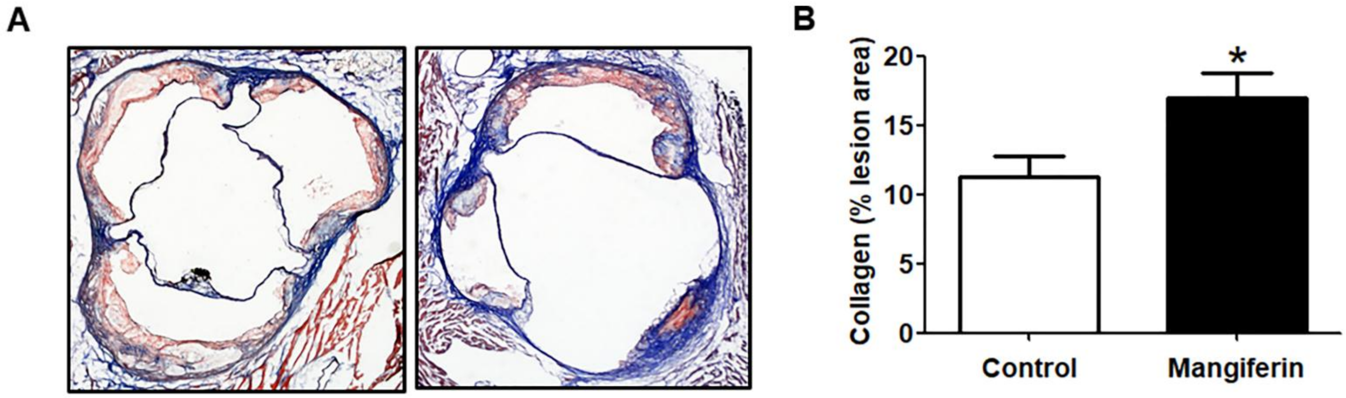

Supplementary Figure 1. Mangiferin increases collagen content in aortic roots of apoE$^{-/-}$ mice. Representative microscopic images (**A**, **B**) and quantification (**C**) of aortic sinus lesions with Masson's trichrome staining. Original magnification: 40×. Values are expressed as the mean ± SEM (n =15/group). *P < 0.05 *vs*. control group.

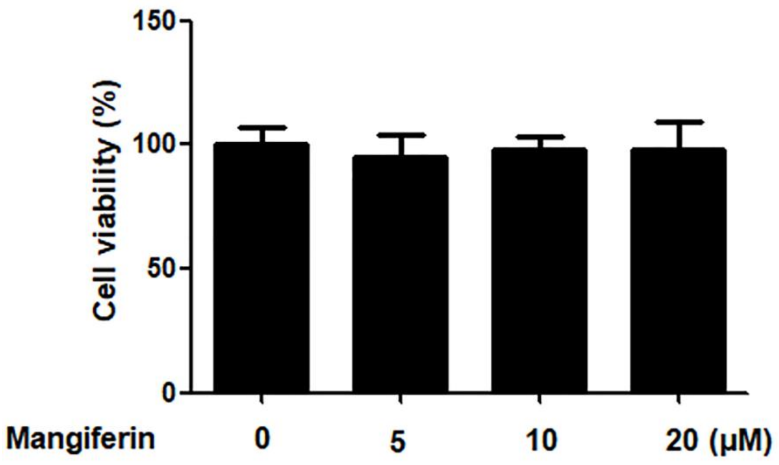

Supplementary Figure 2. The cytotoxic effects of mangiferin on RAW264.7 macrophage-derived foam cells. RAW264.7 macrophage-derived foam cells were exposed to different concentrations of mangiferin (0, 5, 10, and 20 μM) for 24 h. Then, the MTT assay was used to detect cell viability. All data are the mean ± SEM (n=3) *vs.* control group.

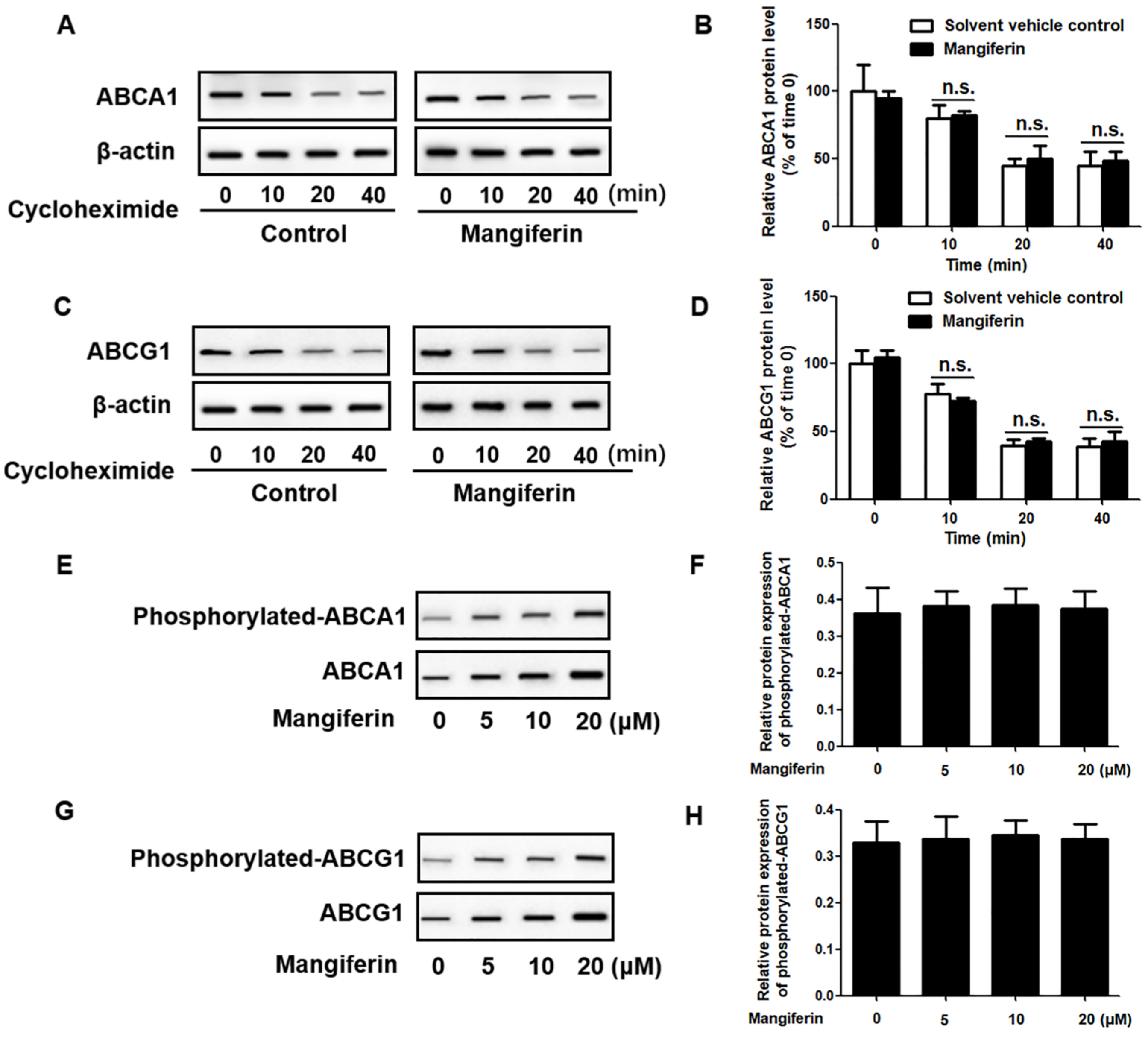

Supplementary Figure 3. Effects of mangiferin on the degradation and phosphorylation of ABCA1/G1 protein. (**A**–**D**) RAW264.7 macrophages were incubated for 24 h with 20 μM mangiferin or an equal amount of the solvent vehicle control (DMSO). Then, the cells were treated with cycloheximide (CHX; 140 μM) and lysed at different time points (0, 10, 20, and 40 min). Western blot analysis was used to monitor the decline in ABCA1/G1 protein levels upon CHX treatment in the presence or absence of mangiferin. All data are the mean ± SEM (n=3) *vs.* control group. (**E**–**H**) RAW264.7 macrophage-derived foam cells were exposed to different concentrations of mangiferin (0, 5, 10, and 20 μM) for 24 h. Then, western blot analysis showed the phosphorylation level of ABCA1/G1. All data are the mean ± SEM (n=3) *vs.* control group.
